# eHealth literacy, cyberchondria and perceived stress among older adults in primary care: a cross-sectional study

**DOI:** 10.1186/s12877-026-07799-8

**Published:** 2026-06-10

**Authors:** Türkan Akyol Güner

**Affiliations:** Zonguldak Bülent Ecevit University, Faculty of Health Sciences, Esenköy, Kozlu, Zonguldak, 67600 Turkey

**Keywords:** eHealth literacy, Cyberchondria, Perceived stress, Older adults, Primary care, Digital health

## Abstract

**Background:**

Digital health information use among older adults has increased rapidly; however, higher exposure to online health information may also contribute to anxiety-related behaviors such as cyberchondria. Evidence regarding the associations among eHealth literacy, cyberchondria, and perceived stress in older adults within primary care settings remains limited. This study aimed to examine the associations between eHealth literacy, cyberchondria, and perceived stress among older adults attending a primary healthcare center and to evaluate how these variables differ according to sociodemographic and health-related characteristics.

**Methods:**

This descriptive cross-sectional study was conducted between March and December 2025 in a Family Health Center in Zonguldak, Türkiye. The sample consisted of 303 adults aged ≥ 65 years who actively used the internet for health information. Data were collected using a Sociodemographic Characteristics Form, the eHealth Literacy Scale (eHEALS), the Cyberchondria Severity Scale (CSS), and the Perceived Stress Scale (PSS-14). Since scale scores were not normally distributed, non-parametric tests (Mann–Whitney U and Kruskal–Wallis) were used. Three linear regression models (unadjusted and adjusted for age, gender, and education level) were constructed to examine the associations among eHealth literacy, cyberchondria, and perceived stress.

**Results:**

Participants demonstrated moderate levels of eHealth literacy, cyberchondria, and perceived stress. eHealth literacy scores were significantly higher among women, younger-old adults, participants with higher education and income levels, and those with more frequent internet use (*p* < 0.05). Cyberchondria levels varied according to age, education, income, chronic disease status, and internet use patterns (*p* < 0.05). Perceived stress was mainly associated with health-related variables. Regression analyses revealed a significant positive association between eHealth literacy and cyberchondria (β = 0.399, *p* < 0.001). Although the overall regression model for perceived stress was not statistically significant (*p* = 0.109), cyberchondria showed a small but significant negative association with perceived stress (β = −0.132, *p* = 0.037).

**Conclusions:**

Higher eHealth literacy among older adults may coexist with increased cyberchondria rather than functioning solely as a protective factor. These findings suggest that digital health competence alone may not reduce anxiety associated with online health information seeking. Primary care interventions should therefore focus not only on improving access to digital health information but also on enhancing critical appraisal skills and supporting anxiety management strategies tailored to older adults.

## Background

Global demographic transformations indicate that the older population is increasing rapidly worldwide and that societies are entering a pronounced aging process. According to the United Nations Population Division’s World Population Prospects 2024, individuals aged 65 years and older currently constitute approximately 9% of the global population, and this proportion is projected to rise to 16% by 2050 [1]. A similar trend is observed in Türkiye; the proportion of individuals aged 65 years and older increased from 8.8% in 2018 to 10.6% in 2024, exceeding a critical threshold, with the older population surpassing 9 million [2].

With aging, the prevalence of chronic diseases increases and physical functioning declines, leading to greater healthcare needs among older adults [3–5]. These changes highlight the growing importance of digital solutions and eHealth applications in healthcare delivery. In the digital era, widespread internet access has enabled individuals to obtain health information at any time. However, concerns regarding the accuracy of online information and the ability of older adults to critically evaluate such information have introduced new challenges [6, 7].

eHealth literacy, defined as the ability to seek, find, understand, evaluate, and apply health information obtained from electronic sources to address health problems, plays a key role in modern health management [8, 9]. For older adults, searching for health information online may provide important advantages, particularly when physical limitations restrict participation in face-to-face education. Nevertheless, age-related cognitive, technological, and socioeconomic barriers may limit eHealth literacy levels within this population [3, 10]. Existing literature suggests that older adults generally have lower eHealth literacy levels compared with younger groups, and that factors such as age, educational level, and internet use experience directly influence this competency [11, 12].

Uncontrolled and intensive use of the internet for health-related purposes has also led to the emergence of a new behavioral phenomenon known as cyberchondria. Cyberchondria refers to excessive and repetitive online searching for disease-related information that increases anxiety levels and may result in self-misdiagnosis [13, 14]. The underlying reassurance-seeking model suggests that individuals turn to the internet to alleviate health-related concerns; however, exposure to conflicting information may intensify anxiety and create a vicious cycle [15, 16]. Cyberchondriac behaviors may lead individuals to excessively monitor bodily sensations, potentially elevating perceived stress levels, which in turn may be associated with physiological symptoms such as headaches and hypertension [17, 18].

Perceived stress refers to the degree to which individuals appraise situations in their lives as unpredictable, uncontrollable, and overwhelming [19]. Among older adults, perceived stress is particularly relevant due to the accumulation of age-related stressors including chronic disease management, functional decline, and social isolation. In the context of digital health behaviors, perceived stress may be both an outcome associated with cyberchondria—arising from repeated exposure to alarming health content—and a factor that influences the extent to which older adults engage in online health information seeking.

Conceptually, these three constructs can be linked through a sequential pathway: older adults with lower eHealth literacy may have greater difficulty critically appraising online health information, which may increase susceptibility to cyberchondria—defined as excessive, anxiety-driven online health information seeking. Elevated cyberchondria, in turn, may be associated with heightened perceived stress through repeated exposure to alarming or contradictory health content. This pathway aligns with the reassurance-seeking model, which posits that individuals search online to reduce health-related uncertainty, yet exposure to inconsistent information may paradoxically amplify anxiety [15, 16]. However, it is also plausible that higher eHealth literacy may increase exposure to health information, thereby elevating cyberchondria risk, suggesting that the direction of this association may be more complex than a simple protective model.

The association between eHealth literacy and cyberchondria remains a debated issue in the literature. Some studies suggest that higher eHealth literacy enables individuals to critically evaluate information, thereby reducing cyberchondria [20], whereas others indicate that individuals with greater digital competence may face increased exposure to health information, which may elevate the risk of cyberchondria [21]. Particularly among older adults with limited digital skills, stress arising from online health information may represent a critical psychological factor associated with overall health management and quality of life [4, 22].

A review of the literature shows that most studies on eHealth literacy and cyberchondria have focused on university students or young adults [6, 20]. Although some research has examined eHealth literacy among older adults, comprehensive studies investigating the associations among digital health literacy, cyberchondria, and perceived stress within this population—particularly in the context of primary healthcare settings—remain limited [9, 10]. Family Health Centers, as the primary level of healthcare delivery, constitute a critical setting where older adults receive preventive care and where anxiety related to digital health information use can be identified [10].

Therefore, this study was designed to address this gap in the literature by examining the associations among eHealth literacy, cyberchondria, and perceived stress among older adults attending a primary healthcare center. The significance of this study lies in revealing how older adults’ capacity to evaluate online health information is associated with their psychological well-being and effective health management [3, 11]. The findings are expected to strengthen the counseling role of primary healthcare professionals working with older adults, support the development of age-friendly digital health policies, and contribute to reducing unnecessary healthcare utilization driven by cyberchondria-related concerns.

## Methods

### Study design

This study was planned as a descriptive, cross-sectional study.

### Setting and time

The study was conducted between 30 March 2025 and 30 December 2025 with older adults registered at a Family Health Center located in the city center of Zonguldak, Türkiye. This center was selected because it served a population with diverse sociodemographic characteristics—including variation in educational attainment (ranging from literate-only to university-educated individuals), income levels (predominantly middle income with representation from low- and high-income groups), and occupational backgrounds (including retired, employed, and unemployed older adults)—and had the highest proportion of registered older adults in the region. As the study was conducted in a single center, the generalizability of the findings to other settings or populations should be interpreted with caution.

### Population and sample

The study population consisted of 689 individuals aged 65 years and older registered at the selected Family Health Center. A convenience sampling method was employed; older adults who attended the center during the data collection period were approached consecutively for eligibility screening. Not all 689 registered individuals were directly invited; rather, the sample was drawn from those who visited the center during the study period. Using the known population sample size formula, the minimum required sample size was calculated as 247 at a 95% confidence level and a 5% margin of error (d = 0.05). Parameters from previous studies examining eHealth literacy and cyberchondria among older adults were considered during sample size estimation [23, 24]. The study was completed with 303 older adults who met the inclusion criteria and agreed to participate voluntarily. The remaining individuals were excluded due to not actively using the internet (*n* = 195), not meeting cognitive assessment criteria (SMMSE < 24; *n* = 72), inability to complete the questionnaire because of visual or hearing impairments (*n* = 55), history of neuropsychiatric disorders (*n* = 23), or refusal to participate (*n* = 41).

Regarding the exclusion of non-internet users (*n* = 195), this restriction was a deliberate methodological decision. Because the constructs under investigation—eHealth literacy and cyberchondria—are inherently dependent on internet use for health information, including non-internet users would have rendered the primary study variables unmeasurable. However, this exclusion introduces selection bias, and the findings are therefore applicable only to internet-using older adults, which is discussed as a limitation.

### Inclusion and exclusion criteria

Inclusion criteria were: being aged 65 years or older, being at least literate, actively using the internet to obtain health information, providing written informed consent, and having normal cognitive functioning as indicated by a score of above 23 (i.e., 24 or higher) on the Standardized Mini-Mental State Examination (SMMSE). Scores of 24 and above are accepted as indicating normal cognitive functioning, whereas scores of 23 and below indicate possible cognitive impairment [25, 26]. Active internet use was operationally defined as using the internet at least once per month for any purpose, including but not limited to health information seeking. This criterion was assessed through self-report during the initial screening interview.

Exclusion criteria included having visual or hearing impairments that prevented understanding or responding to the questionnaire, having a diagnosed neuropsychiatric disorder such as dementia or Alzheimer’s disease, or withdrawing from the study at any stage.

### Data collection instruments

Data were collected using a Sociodemographic Characteristics Form, the eHealth Literacy Scale, the Cyberchondria Severity Scale, and the Perceived Stress Scale.

#### Sociodemographic characteristics form

A 12-item questionnaire developed by the researcher based on the literature was used [4, 9–11, 27]. The form was reviewed by three experts in gerontological nursing and public health for content validity, and a pilot study was conducted with 15 older adults (not included in the final sample) to assess clarity and comprehensibility. The form included questions on age, gender, marital status, education, employment status, income level, general health perception, presence of chronic disease, regular medication use, methods of accessing health information, internet use frequency, and devices used for internet access. Income level was categorized based on self-reported subjective assessment as low, middle, or high. Internet use frequency was categorized as at least once daily, every few days, once per week, or 1–2 times per month.

#### eHealth literacy scale (eHEALS)

Developed by Norman and Skinner (2006) [8]. Turkish adaptation by Gencer (2017) [28]. Eight items, 5-point Likert scale –, , total scores 8–40; higher scores indicate higher eHealth literacy. Cronbach’s α values were 0.89 for the original scale, 0.86 for the Turkish adaptation, and 0.90 in the present study, indicating good internal consistency. Although originally designed for general adult populations, eHEALS has been validated across age groups including older adults [24]. However, the instrument may not fully capture digital challenges specific to older adults, which is noted as a limitation.

#### Cyberchondria severity scale (CSS)

Developed by McElroy and Shevlin (2014) [29]. Turkish adaptation by Uzun (2021) [30]. Thirty-three items, 5-point Likert scale. The CSS is a continuous measure without established clinical cut-off scores; higher total scores indicate higher levels of cyberchondria, and results are interpreted in terms of relative severity within the sample. Cronbach’s α values were 0.94 for the original scale, 0.96 for the Turkish adaptation, and 0.92 in the present study, indicating excellent internal consistency. 

#### Perceived stress scale (PSS-14)

Developed by Cohen et al. (1983) [19]. Turkish adaptation by Eskin et al. (2013) [31]. Fourteen items, 5-point Likert scale (0–4). Higher scores indicate higher perceived stress. Seven positively stated items are reverse scored. Cronbach’s α values were 0.84 for both the original scale and the Turkish adaptation, and 0.88 in the present study, indicating good internal consistency. 

#### Standardized mini mental state examination (SMMSE)

Developed by Folstein et al. (1975) [25]. Turkish standardized version by Güngen et al. (2002) [26]. Five subdomains, total 30 points. Scores of 24 and above indicate normal cognitive functioning. The SMMSE was administered by the researcher (a trained nursing faculty member with experience in geriatric research) in a quiet room within the Family Health Center, prior to administering the study questionnaires.

### Statistical analysis

Statistical analyses were performed using IBM SPSS Statistics version 25.0. Descriptive statistics were presented as frequencies and percentages for categorical variables, and as medians with interquartile ranges (IQR) for continuous variables. Normality was assessed using the Shapiro–Wilk test. Non-parametric tests were applied: Mann–Whitney U for two-group and Kruskal–Wallis for multi-group comparisons, with Bonferroni-adjusted post hoc analyses. Effect sizes were calculated as r = Z / √N for Mann–Whitney U tests and η² = H / (*N* − 1) for Kruskal–Wallis tests, interpreted according to Cohen’s (1992) guidelines [32].

Three separate linear regression models were constructed. Model 1 examined eHealth literacy as a predictor of cyberchondria. Model 2 examined eHealth literacy and cyberchondria as predictors of perceived stress. Model 3 examined cyberchondria and perceived stress as predictors of eHealth literacy. Perceived stress was not included in Model 1 because the conceptual framework positions perceived stress as a potential downstream correlate rather than a predictor of cyberchondria. Both unadjusted models and models adjusted for age, gender, and education level as potential confounders were estimated. Linear regression was justified given the adequate sample size (*n* = 303) and the robustness of regression to moderate normality deviations in large samples [33]. Multicollinearity was assessed using VIF; all values were below 2.0. Cohen’s f² effect sizes were calculated. With 303 participants and up to two predictors, the study had adequate power (> 0.95) to detect medium effect sizes (f² = 0.15) at α = 0.05 [32]. Statistical significance was set at *p* < 0.05.

### Ethical considerations

Ethical approval was obtained from the Zonguldak Bülent Ecevit University Human Research Ethics Committee (Approval No: 11.03.2025–573724). Face-to-face interviews were conducted by the researcher, lasting approximately 20–25 min each. Written informed consent was obtained from all participants. All collected data were stored securely in a password-protected database accessible only to the researcher. Participant identifiers were removed prior to data entry to ensure anonymity and confidentiality.

## Results

The study included a total of 303 community-dwelling older adults. As presented in Tables 1, 53.47% of the participants were male and 46.53% were female. The largest age group was 65–70 years (57.10%). Most participants were married (71.29%) and had primary school education (32.34%). The majority were retired (60.07%) and reported a middle income level (67.33%).

**Table 1:** Sociodemographic characteristics of the participants (*N* = 303)

Variable	*n* (%)
Gender	
Male	162 (53.47)
Female	141 (46.53)
Age (years)	
65–70	173 (57.10)
71–76	71 (23.43)
≥76	59 (19.47)
Marital status	
Married	216 (71.29)
Widowed	58 (19.14)
Divorced	15 (4.95)
Single	14 (4.62)
Education level	
Primary school	98 (32.34)
High school	64 (21.12)
Middle school	61 (20.13)
Literate	42 (13.86)
University	38 (12.54)
Employment status	
Retired	182 (60.07)
Not working	90 (29.70)
Working	31 (10.23)
Income level	
Middle	204 (67.33)
Low	85 (28.05)
High	14 (4.62)

Percentages are calculated based on the total sample (*N* = 303)

As shown in Table 2, most participants perceived their general health status as moderate (74.26%). The majority had at least one chronic disease (76.57%) and reported regular medication use (81.52%). Mobile phones were the primary device for internet access (93.73%), and most participants used the internet at least once daily (68.65%).

**Table 2:** Health characteristics and internet use for health information among participants (*N* = 303)

Variable	*n* (%)
General health status	
Moderate	225 (74.26)
Good	50 (16.50)
Poor	28 (9.24)
Chronic disease	
Yes	232 (76.57)
No	71 (23.43)
Regular medication use	
Yes	247 (81.52)
No	56 (18.48)
Use of the internet for health information	
Yes	233 (76.90)
No	70 (23.10)
Device used for internet access	
Mobile phone	284 (93.73)
Computer	19 (6.27)
Frequency of internet use	
At least once daily	208 (68.65)
1–2 times per month	42 (13.86)
Every few days	39 (12.87)
Once per week	14 (4.62)

Percentages are calculated based on the total sample (*N* = 303)

As shown in Table 3, the Shapiro–Wilk test indicated that all scale scores significantly deviated from normality (*p* < 0.001). The median eHEALS score was 25.50 (IQR = 15.00), the median CSS score was 65.00 (IQR = 33.00), and the median PSS-14 score was 28.00 (IQR = 4.00). Overall, these findings indicate moderate levels of eHealth literacy, cyberchondria, and perceived stress among the participants.

**Table 3:** Descriptive statistics and normality test results of the study scales

Scale	*N*	Mean	Median	IQR	Skewness	Kurtosis	S-W *p*
eHEALS	303	24.58	25.50	15.00	−0.388	−0.546	< 0.001
CSS	303	72.20	65.00	33.00	0.627	0.141	< 0.001
PSS-14	303	26.28	28.00	4.00	−1.238	3.848	< 0.001

*eHEALS*  eHealth Literacy Scale, *CSS*  Cyberchondria Severity Scale, *PSS-14*  Perceived Stress Scale-14, *IQR*  Interquartile range

S-W *p* = Shapiro–Wilk test *p*-value

As shown in Tables 4, eHealth literacy scores differed significantly according to several sociodemographic and health-related variables. Higher eHEALS scores were observed among women (*r* = 0.19), younger age groups (η² = 0.14), participants with higher education levels (η² = 0.25), those who were currently working, and older adults reporting better general health status (*p* < 0.05). Post-hoc analyses indicated that individuals without chronic disease had significantly higher eHEALS scores than those with chronic disease, and older adults not using regular medication had significantly higher scores.

**Table 4:** Comparison of eHealth Literacy Scale (eHEALS) scores according to sociodemographic and health-related characteristics

Variable	eHEALS Median (IQR)	Test statistic	*p*	Effect size	Post-hoc
Gender					
Female	28 (8–40)	Z = 13206.500	**0.003**	*r* = 0.19	Female > Male
Male	24 (8–40)				
Age group					
65–70	28 (8–40)	K = 43.551	**< 0.001**	η²=0.14	65–70 > 71–75 > ≥ 76
71–75	24 (8–40)				
≥76	16 (8–40)				
Marital status					
Married	28 (8–40)	K = 34.899	**< 0.001**	η²=0.12	Married > Widowed
Single	27 (8–40)				
Divorced	27 (8–32)				
Widowed	17 (8–40)				
Education level					
Literate	16 (8–30)	K = 75.210	**< 0.001**	η²=0.25	University > Literate, Primary, Middle
Primary school	24 (8–40)				
Middle school	26 (8–40)				
High school	29 (8–40)				
University	32 (14–40)				
Employment status					
Working	32 (8–40)	K = 19.775	**< 0.001**	η²=0.07	Working > Not working
Not working	24 (8–33)				
Retired	26 (8–40)				
Income level					
Low	25 (8–40)	K = 6.287	**0.043**	η²=0.02	High > Low
Middle	24 (8–40)				
High	28 (14–40)				
General health status					
Poor	24 (8–40)	K = 8.036	**0.018**	η²=0.03	Good > Poor, Moderate
Moderate	24 (8–40)				
Good	30 (8–40)				
Chronic disease					
Yes	24 (8–40)	Z = 5860.000	**0.001**	*r* = 0.19	No > Yes
No	28 (8–40)				
Regular medication use					
Yes	24 (8–40)	Z = 4926.500	**0.001**	*r* = 0.19	No > Yes
No	29 (12–40)				
Internet use for health info					
Yes	28 (8–40)	Z = 13953.000	**< 0.001**	*r* = 0.35	Yes > No
No	16 (8–32)				
Internet use frequency					
At least once daily	28 (8–40)	K = 64.028	**< 0.001**	η²=0.21	Daily > Weekly, Monthly
Every few days	24 (8–32)				
Once per week	16 (8–32)				
1–2 times/month	14 (8–31)				

Effect sizes: r = Z / √N; η² = H / (*N* − 1). Bold p-values indicate statistical significance (*p* < 0.05). Post-hoc comparisons are Bonferroni-adjusted

*Z* Mann–Whitney U test, *K* Kruskal–Wallis test

Cyberchondria scores also varied across multiple characteristics. Significant differences were identified according to age group, marital status, education level, income level, presence of chronic disease, regular medication use, and internet-related variables (*p* < 0.05). Notably, single older adults had significantly higher CSS scores than married and widowed individuals. Older adults with chronic disease had significantly higher cyberchondria scores than those without (Table 5).

**Table 5:** Comparison of Cyberchondria Severity Scale (CSS) scores according to sociodemographic and health-related characteristics

Variable	CSS Median (IQR)	Test statistic	*p*	Effect size	Post-hoc
Gender					
Female	68 (33–139)	Z = 10932.000	0.910	*r* = 0.01	—
Male	65 (33–149)				
Age group					
65–70	61 (33–132)	K = 13.862	**< 0.001**	η²=0.05	≥ 76 > 65–70
71–75	67 (33–130)				
≥76	68 (33–149)				
Marital status					
Married	65 (33–139)	K = 9.850	**0.020**	η²=0.03	Single > Married, Widowed
Single	98 (41–132)				
Divorced	67 (55–149)				
Widowed	66 (33–128)				
Education level					
Literate	56 (33–149)	K = 20.275	**< 0.001**	η²=0.07	University > Literate
Primary school	65 (33–139)				
Middle school	65 (33–115)				
High school	75 (33–138)				
University	80 (53–132)				
Employment status					
Working	74 (33–149)	K = 4.176	0.124	η²=0.01	—
Not working	66 (33–139)				
Retired	65 (33–138)				
Income level					
Low	79 (33–149)	K = 12.225	**0.002**	η²=0.04	Low > Middle
Middle	65 (33–130)				
High	88 (53–104)				
General health status					
Poor	74 (33–130)	K = 0.043	0.979	η²<0.01	—
Moderate	66 (33–138)				
Good	65 (33–149)				
Chronic disease					
Yes	83 (33–138)	Z = 5338.500	**< 0.001**	*r* = 0.22	Yes > No
No	65 (33–149)				
Regular medication use					
Yes	80 (33–138)	Z = 5378.500	**0.016**	*r* = 0.14	Yes > No
No	65 (33–149)				
Internet use for health info					
Yes	68 (41–149)	Z = 12313.000	**< 0.001**	*r* = 0.28	Yes > No
No	53 (33–98)				
Internet use frequency					
At least once daily	80 (33–126)	K = 23.220	**< 0.001**	η²=0.08	Weekly > Daily, Monthly
Every few days	76 (33–119)				
Once per week	65 (33–149)				
1–2 times/month	52 (33–104)				

Effect sizes: r = Z / √N; η² = H / (*N* − 1). Bold p-values indicate statistical significance (*p* < 0.05). Post-hoc comparisons are Bonferroni-adjusted

*Z*  Mann–Whitney U test, *K*  Kruskal–Wallis test

Perceived stress scores showed fewer significant differences across groups. Significant variations were observed according to general health status, chronic disease presence, and regular medication use (*p* < 0.05). Post-hoc analyses indicated that older adults reporting poor health had significantly higher PSS-14 scores than those reporting good health, and participants with chronic disease had significantly higher perceived stress than those without (Table 6).

**Table 6:** Comparison of Perceived Stress Scale (PSS-14) scores according to sociodemographic and health-related characteristics

Variable	PSS-14 Median (IQR)	Test statistic	*p*	Effect size	Post-hoc
Gender					
Female	28 (0–41)	Z = 10255.500	0.290	*r* = 0.06	—
Male	28 (0–41)				
Age group					
65–70	28 (0–41)	K = 2.282	0.319	η²<0.01	—
71–75	28 (9–35)				
≥76	28 (6–36)				
Marital status					
Married	28 (0–40)	K = 3.045	0.385	η²=0.01	—
Single	28 (23–38)				
Divorced	27 (14–32)				
Widowed	28 (0–41)				
Education level					
Literate	28 (0–41)	K = 5.916	0.205	η²=0.02	—
Primary school	28 (0–41)				
Middle school	28 (21–35)				
High school	28 (6–40)				
University	26 (11–39)				
Employment status					
Working	28 (14–38)	K = 1.587	0.452	η²<0.01	—
Not working	28 (7–41)				
Retired	28 (0–41)				
Income level					
Low	27 (6–41)	K = 2.816	0.245	η²<0.01	—
Middle	28 (0–41)				
High	26 (11–38)				
General health status					
Poor	28 (14–39)	K = 18.347	**< 0.001**	η²=0.06	Poor > Good
Moderate	27 (0–41)				
Good	26 (6–32)				
Chronic disease					
Yes	28 (0–41)	Z = 10287.000	**< 0.001**	*r* = 0.21	Yes > No
No	26 (6–40)				
Regular medication use					
Yes	28 (0–41)	Z = 8641.500	**< 0.001**	*r* = 0.22	Yes > No
No	26 (6–40)				
Internet use for health info					
Yes	28 (0–40)	Z = 6422.000	0.311	*r* = 0.06	—
No	28 (13–41)				
Internet use frequency					
At least once daily	28 (6–41)	K = 3.337	0.503	η²=0.01	—
Every few days	28 (15–40)				
Once per week	25 (0–41)				
1–2 times/month	28 (0–36)				

Effect sizes: r = Z / √N; η² = H / (*N* − 1). Bold *p*-values indicate statistical significance (*p* < 0.05)

*Z*  Mann–Whitney U test, *K*  Kruskal–Wallis test

— = not applicable or not significant

Linear regression analyses examined the associations among eHealth literacy, cyberchondria, and perceived stress (Table 7). In the unadjusted Model 1, eHealth literacy significantly predicted cyberchondria (β = 0.399, *p* < 0.001, 95% CI [0.811, 1.389]), explaining approximately 15.9% of the variance (Cohen’s f² = 0.189). This association remained significant after adjusting for age, gender, and education (β = 0.342, *p* < 0.001). In the unadjusted Model 2, the overall model was not statistically significant (F = 2.23, *p* = 0.109, f² = 0.015). However, cyberchondria showed a small but statistically significant negative association with perceived stress (β = −0.132, *p* = 0.037, 95% CI [− 0.060, − 0.002]). In Model 3, cyberchondria significantly predicted eHealth literacy (β = 0.403, *p* < 0.001, f² = 0.190). After adjusting for covariates, age and education emerged as significant predictors of eHealth literacy (both *p* < 0.001).

**Table 7:** Linear regression analyses examining the associations among eHealth literacy, cyberchondria, and perceived stress

Model	Predictor	B	SE	β	t	95% CI	*p*	*R*²	Adj *R*²	F (Model *p*)	f²
1a	eHEALS	1.100	0.147	0.399	7.49	[0.811, 1.389]	**< 0.001**	0.159	0.156	56.10 (< 0.001)	0.189
1b	eHEALS	0.944	0.162	0.342	5.83	[0.626, 1.262]	**< 0.001**	0.198	0.188	18.43 (< 0.001)	0.247
	Age	0.312	0.148	0.118	2.11	[0.021, 0.603]	**0.036**				
	Gender	−1.204	2.882	−0.023	−0.42	[− 6.877, 4.469]	0.677				
	Education	1.358	1.221	0.068	1.11	[− 1.044, 3.760]	0.267				
2a	eHEALS	0.023	0.041	0.036	0.57	[− 0.057, 0.103]	0.567	0.015	0.008	2.23 (0.109)	0.015
	CSS	−0.031	0.015	−0.132	−2.09	[− 0.060, − 0.002]	**0.037**				
2b	eHEALS	0.018	0.044	0.028	0.41	[− 0.068, 0.104]	0.682	0.035	0.019	2.16 (0.058)	0.036
	CSS	−0.028	0.015	−0.119	−1.83	[− 0.058, 0.002]	0.068				
	Age	0.021	0.040	0.034	0.53	[− 0.058, 0.100]	0.599				
	Gender	−0.346	0.784	−0.027	−0.44	[− 1.889, 1.197]	0.660				
	Education	−0.492	0.332	−0.100	−1.48	[− 1.146, 0.162]	0.140				
3a	CSS	0.146	0.019	0.403	7.50	[0.108, 0.184]	**< 0.001**	0.160	0.155	28.15 (< 0.001)	0.190
	PSS-14	0.047	0.083	0.031	0.57	[− 0.116, 0.210]	0.567				
3b	CSS	0.121	0.020	0.334	5.98	[0.081, 0.161]	**< 0.001**	0.307	0.296	26.39 (< 0.001)	0.443
	PSS-14	0.031	0.078	0.020	0.40	[− 0.122, 0.184]	0.692				
	Age	−0.682	0.114	−0.312	−5.97	[− 0.907, − 0.457]	**< 0.001**				
	Gender	1.032	0.954	0.054	1.08	[− 0.845, 2.909]	0.281				
	Education	1.724	0.404	0.233	4.27	[0.930, 2.518]	**< 0.001**				

*B*  Unstandardized coefficient, *SE*  Standard error, *β*  Standardized coefficient, *CI*  Confidence interval, *f*² Cohen’s f² effect size, *DV*  Dependent variable

Models a = unadjusted; Models b = adjusted for age, gender, and education level

Bold *p*-values indicate statistical significance (*p* < 0.05)

## Discussion

This study examined the associations among eHealth literacy, cyberchondria, and perceived stress among older adults attending a primary care center. The findings indicate that levels of eHealth literacy, cyberchondria, and perceived stress were generally moderate in this population.

The moderate level of eHealth literacy is consistent with previous research in primary care settings among older adults [10, 11]. Salar and Duran (2023) similarly reported moderate eHEALS scores among older adults in Turkish Family Health Centers, while Shi et al. (2023) found comparable levels in a systematic review of Chinese older adults. These findings suggest that although access to digital health information is increasing, effective appraisal and application of online health information may still be limited.

The moderate level of cyberchondria observed among participants indicates that online health information-seeking behaviors may extend beyond simple information acquisition. It is important to distinguish between cyberchondria—which captures health-specific anxiety driven by online information seeking—and general perceived stress, which reflects broader appraisals of life circumstances. The finding that cyberchondria was negatively, albeit weakly, associated with perceived stress (β = −0.132, *p* = 0.037) may appear counterintuitive. However, this may suggest that older adults who engage in more active online health information seeking perceive a greater sense of control over their health concerns, potentially reducing general stress perceptions despite elevated health-specific anxiety. Xu and Starcevic (2025) found similar patterns, noting that cyberchondria operated through health-specific cognitive mechanisms rather than generalized stress pathways.

eHealth literacy varied according to sociodemographic and clinical characteristics, supporting the notion that digital health competence is a multidimensional construct. Higher eHealth literacy among women aligns with studies suggesting gender-related differences in health information-seeking behaviors [34]. This is consistent with Çetin et al. (2025), who reported higher eHEALS scores among women. Higher scores among younger-old adults and those with higher education and income levels may reflect differences in digital skills and critical evaluation capacity [4, 9].

Cyberchondria also differed across sociodemographic and clinical variables. Higher levels among older age groups, older adults with chronic illness, and those with poorer self-rated health suggest that health-related uncertainty may intensify online reassurance-seeking behaviors [13, 29, 35]. Previous research in the same region demonstrated that health literacy among individuals with diabetes was insufficient and strongly associated with rational drug use, underscoring the critical role of literacy competencies in chronic disease management [5]. Lai et al. (2025) [36] similarly reported that chronic disease patients exhibited higher cyberchondria levels. The finding that single older adults exhibited significantly higher cyberchondria levels than married and widowed individuals is notable. Single older adults may lack informal health information gatekeepers—such as spouses—who could provide reassurance or encourage direct consultation with healthcare providers. Social isolation, more prevalent among unmarried older adults, may increase reliance on online sources for health reassurance. The absence of shared health management experiences may intensify health-related uncertainty and drive more frequent online searching [35, 37].

Perceived stress was more strongly associated with health-related variables than with sociodemographic factors [38–40]. Polat et al. (2022) found a significant positive relationship between cyberchondria and perceived stress in young adults, contrasting with the present findings. This age-related discrepancy may suggest that the mechanisms linking cyberchondria to stress differ across age groups: younger adults may experience cyberchondria as an additional stressor, whereas older adults—facing multiple competing health stressors—may perceive online health information seeking as one of many coping strategies rather than a primary stress source.

Regression analyses revealed a moderate positive association between eHealth literacy and cyberchondria. This finding is consistent with Mansur and Ciğerci (2022) and Özişli and Ağcadağ (2022) in Turkish samples, while contrasting with earlier findings in younger adult populations. These findings suggest a positive cross-sectional association between digital competence and anxiety-driven search behavior, though the cross-sectional design precludes causal interpretation.

From a primary care perspective, Family Health Centers are ideal settings for identifying problematic online health information-seeking behaviors. The integration of brief digital health literacy screening tools (e.g., eHEALS) into routine geriatric assessments may help identify older adults at risk of cyberchondria. Structured patient education modules on evaluating online health information credibility, and training primary care professionals to recognize signs of cyberchondria, could reduce unnecessary healthcare utilization.

## Conclusion

This study demonstrated that older adults attending primary care services exhibit moderate levels of eHealth literacy, cyberchondria, and perceived stress. A positive association was identified between eHealth literacy and cyberchondria. Although the overall regression model for perceived stress was not significant, cyberchondria showed a small negative association with perceived stress, suggesting differential mechanisms for health-specific and generalized stress among older adults. These findings suggest that increased access to digital health information does not always function as a protective factor. Interventions should emphasize critical appraisal skills and anxiety management. Future longitudinal studies are needed to clarify temporal pathways and develop age-sensitive digital health strategies.

### Strengths and limitations

A major strength of this study is that it was conducted in a primary care setting with community-dwelling older adults. The study examined eHealth literacy, cyberchondria, and perceived stress together within the same analytical framework.

However, several limitations should be considered. First, the cross-sectional design limits causal or temporal inferences. Second, the single-center design in Zonguldak may limit generalizability. Third, restricting the sample to internet-using older adults (excluding *n* = 195 non-users) introduces selection bias. Fourth, self-report measures are subject to social desirability and recall bias. Fifth, eHEALS may not fully capture digital health skills specific to older adults, and the CSS lacks clinical cut-off scores. Sixth, although adjusted regression models controlled for key confounders, residual confounding from unmeasured variables cannot be ruled out. Finally, the convenience sampling method may introduce additional selection bias.

## Data Availability

The datasets generated and/or analysed during the current study are available from the corresponding author on reasonable request.
